# Regulatory Performance of African National Medicines Regulatory Authorities Achieving WHO Maturity Level 3: Identifying Best Practices

**DOI:** 10.1007/s43441-025-00879-8

**Published:** 2025-10-27

**Authors:** Mercy Owusu-Asante, Delese Mimi Darko, Boitumelo Semete-Makokotieia, Christianah Mojisola Adeyeye, Adam Mitangu Fimbo, Richard Rukwata, Ghada Zaki, Stuart Walker, Sam Salek

**Affiliations:** 1 School of Health, Medicine and Life Sciences, University of Hertfordshire, Hatfield, Herts, AL10 9AB UK; 2 Food and Drugs Authority, Accra, Ghana; 3 South African Health Products Regulatory Authority, Pretoria, South Africa; 4 National Agency for Food and Drug Administration and Control of Nigeria, Abuja, Nigeria; 5 Tanzania Medicines and Medical Devices Authority, Dar es Salaam, Tanzania; 6 Medicines Control Authority of Zimbabwe, Harare, Zimbabwe; 7 Egyptian Drug Authority, Cairo, Egypt; 8 Institute of Global Health and Human Ecology, The American University in Cairo, Cairo, Egypt; 9 Centre for Innovation in Regulatory Science, London, UK; 10 Institute of Medicines Development, Cardiff, UK

**Keywords:** World Health Organization Global Benchmarking Tool (WHO GBT), African national medical regulatory authorities (NMRAs), Regulatory best practices

## Abstract

**Background:**

The World Health Organization (WHO) developed the WHO Global Benchmarking Tool (GBT) to assess and benchmark the drug regulatory systems and practices in national medicines regulatory authorities (NMRAs). The objective of the study was to identify strengths and opportunities for improvement by comparing the regulatory performance of the NMRAs in Egypt, Ghana, Nigeria, South Africa, Tanzania and Zimbabwe, all which have attained maturity level 3 status for medicines and /or vaccines, in order to enhance regulatory review processes and patients’ access to medicines and/or vaccines.

**Methods:**

The NMRAs selected for the study completed a questionnaire that collected data and metrics that facilitated comparative studies among the NMRAs.

**Results:**

The comparative study showed that similarities among these authorities also translated into their strengths. The study revealed that the human resource capacity in African NMRAs is inadequate to fully execute regulatory mandates. Review process map comparison revealed the important observation that these NMRAs conducted labelling review early in the review process rather than in the latter stages of the process.

**Conclusion:**

The study has identified the regulatory best practices that led to the NMRAs achieving WHO GBT maturity level 3. The African Medicines Agency should engage these maturity level-3 NMRAs to explore ways of benefiting from their experience and resources. It is hoped that through such engagement, the NMRAs will be encouraged to further develop their capacity to help the AMA to achieve its mandate. Additionally, by addressing the identified gaps and recommendations in the study these NMRAs can achieve WHO GBT maturity level 4 whilst NMRAs who have not yet reached GBT maturity level 3 can also benefit from this study in order to reach higher maturity levels.

## Introduction

Governments have encouraged national medicines regulatory authorities (NMRAs) to benchmark themselves to satisfy stakeholders in public health that these institutions are being efficient, effective and transparent in executing their mandate to ensure the safety, quality and efficacy of medicines and medical products.

According to Magd and Curry “Benchmarking involves learning about your own practices, the best practices of others and then making a change for improvement that will enable you to meet or be the best in the world.” [1]. This definition is supported by others [2, 3]. The World Health Organization (WHO) has stated that “regulatory system benchmarking implies a structured and documented process by which Member States can identify and address gaps with the goal of reaching a level of regulatory oversight commensurate with a stable, well-functioning and integrated regulatory system.” [3] As part of the efforts to strengthen the regulatory systems on a global scale, the WHO developed the Global Benchmarking Tool (GBT). The GBT ranks NMRAs with regard to the maturity level of the regulatory system on a scale of 1 (lowest maturity level) to 4 (highest maturity level) [4] across core regulatory functions. These core regulatory functions, which are applicable to medicines are national regulatory system, registration and marketing authorization, vigilance, market surveillance and control, licensing establishments, regulatory inspection, laboratory testing, clinical trials oversight and national regulatory authority lot release applicable to biological products [3].

Vaz and colleagues recently noted that in addition to inefficient regulatory systems, “the lack of maturity of the regulatory systems for medical products,” impedes timely access to medicines. During the launch of the WHO plan “Delivering Quality-assured Medical Products for All 2019–2023,” the Assistant Director General for Medicines and Health Products established the link between access to quality medicines and the strength of an NMRA. The comment was that “true access and the health gains that come with it can only be achieved if globally, regionally and nationally, health products do what they are meant to do – prevent illness and improve people’s health. They can only do that if sound regulatory systems are in place” [4]. The WHO also reported that NMRAs in developing countries have inadequate resources to regulate new active substances to be used for non-communicable diseases that are becoming prevalent in these countries, apart from being inadequately prepared to manage pandemics through the deployment of facilitated regulatory pathways [4, 5].

As of June 2024, six NMRAs in Africa have been listed as operating at maturity level 3 for medicines and/or vaccines, meaning that these authorities have “stable, well-functioning and integrated regulatory systems” [6.7]. These NMRAs include: the Egyptian Drug Authority (EDA) vaccines; the Food and Drugs Authority of Ghana (FDA) medicines and vaccines; the National Agency for Food and Drug Administration and Control of Nigeria (NAFDAC) medicines and vaccines; the South African Health Products Regulatory Authority (SAHPRA) vaccines; the Tanzania Medicines and Medical Devices Authority (TMDA) medicines and vaccines; and the Medicines Control Authority of Zimbabwe (MCAZ) medicines and vaccines [6, 7].

The EDA (Egypt) joined the International Council for Harmonisation of Technical Requirements for Pharmaceuticals for Human Use (ICH) as an observer in November 2021 and became a full member of ICH in June 2023, marking a significant milestone as the first regulatory member from Africa [8]. This is in line with keeping up with the trend of improving the national medicines regulatory systems in Africa. It may be of interest to note that the ICH has listed the regional harmonization initiatives of the East African Community (EAC) and the Southern African Development Community (SADC) as observers [9].

In 2014, the pharmaceutical markets in South Africa, Egypt, Algeria, Morocco and Nigeria were listed as the major markets in Africa, with a total market value of 70% [10]. The benefits and importance of the relevant NMRAs when the NMRAs are listed by either the WHO or the ICH is a way of satisfying and enhancing stakeholders’ and public interest in these NMRAs.

Guzman and colleagues (2020) reported that the WHO Global benchmarking tool facilitates transparency and confidence in the NMRAs as a result of their assessment by the WHO. Indeed, “the benchmarking tool provides a systematic approach for measuring and strengthening regulatory system capacity to a defined maturity level” [11]. Other benefits that were also reported by Guzman and colleagues included “regulatory reliance and harmonization, timely access to quality-assured medicines and boost to pharmaceutical trade” [11]. To add to all the above “as more countries benchmark their NMRAs, neighboring countries will be encouraged to invest in regulatory systems strengthening” [11].

For NMRAs to benefit from benchmarking, these institutions should have a quality agenda or a benchmarking culture in place to continually improve their quality management systems by incorporating lessons from other institutions who have been proven to be comparatively more successful in providing efficient and effective services to the public and stakeholders [1]. Although access to regulatory data from some NMRAs may be a challenge; a risk-based framework can be used to identify the inadequacies present in a drug regulatory system [2].

According to a recent publication, less mature NMRAs that study more mature NMRAs within their region, improved their regulatory systems [11]. This is a very significant finding and should serve as an important platform to launch positive reforms in the regulatory landscape in the African region.

As the NMRAs in Africa that have achieved maturity level-3 status strive to achieve maturity level 4, such has been accomplished by Saudi Arabia, the Republic of Korea and Singapore [12], it is timely to conduct a comparative study to identify similarities and differences that exist in the regulatory systems of these level-3 NMRAs.

### Study Objectives

The objectives of this study were to identify and compare the best practices from the African NMRAs operating at WHO GBT level 3 that should be implemented by other NMRAs as they strive to achieve WHO GBT higher maturity levels.

## Methods

### Study Participants

The EDA (Egypt), FDA (Ghana), NAFDAC (Nigeria), SAHPRA (South Africa), TMDA (Tanzania) and MCAZ (Zimbabwe), which have been listed as NMRAs operating at maturity level 3 were selected for this study.

### Data Collection Process

To facilitate comparison among the African NMRAs, each authority except for the EDA completed the Optimising Efficiencies in Regulatory Agencies (OpERA) questionnaire, which was designed by the Centre for Innovation in Regulatory Science (CIRS) [13], to collect data and metrics for the regulatory review process in the same manner. Data for the EDA was collected and organized by a senior EDA staff member from publicly accessible domain information on the EDA website.

The OpERA questionnaire, which is an established, standardized, and validated tool is organized into the following six modules:

Module 1: Organization of the authority—relating to the structure, organization, and resources.

Module 2: Types of review models—relating to the review models used for scientific assessment of marketing authorization applications.

Module 3: Key milestones in the review process—relating to the process map and key milestone dates to facilitate review of timelines.

Module 4: Good review practices (GRevP): building quality into the regulatory process—relating to measures that have been implemented to achieve transparency, consistency, and timeliness in the regulatory process.

Module 5: Quality decision-making processes—relating to measures that have been implemented to ensure that decisions that are made are in line with best practice.

Module 6: Concluding observations—relating to the strengths and challenges from the view of the authority in carrying out its mandate.

### Data Processing and Analysis

The qualitative data that was generated through completion of the OpERA questionnaire by the study participants were analyzed using both content analysis as well as frequencies and counts and then reported as such in tables and figures. The quantitative data was processed using Excel and analyzed using descriptive statistics.

## Results

For the purpose of clarity, the results are presented in six parts as follows; Part 1: Organization of the authority; Part 2: Types of review models; Part 3: Key milestones in the review process; Part 4: Good review practices (GRevP); Part 5: Quality decision-making processes, and Part 6: Concluding observations.

### Part 1: Organization of the Authorities

All the authorities except for FDA Ghana, are organized as autonomous authorities to regulate medical products for human and veterinary use, medical devices, and diagnostics. The scope of regulatory activities includes; marketing authorizations/product licenses, clinical authorization, post-marketing surveillance, regulation of advertising, laboratory analysis of samples and regulatory site inspections/visits. Additionally, among other activities, the EDA manages medicine pricing, pharmaceutical establishment licensing, lot release, importation approvals and plans, and customs release in Egypt.

The staff to population ratio ranges from 1.76 staff per million (Tanzania) to 30 per million (Egypt). The authorities are generally funded from two main sources, namely application fees and government contribution. The financial contribution from government to the NMRAs varies from 12% (Tanzania), 22% (Ghana and Nigeria) to 70% (South Africa). Similarly, EDA is funded from two main sources: application fees and government contribution; however, the specific percentage of Egypt's budget allocated to the EDA is not explicitly detailed in the publicly accessible information. In Zimbabwe, the authority is self-funded entirely from fees.

### Part 2: Types of Review Models

The authorities mostly employ the three types of review models for the scientific assessment of medicines; the exceptions apply to Tanzania and Nigeria, which use two of the review models (Table 1). Type 1 (verification) is used by the authorities for WHO-Prequalified products and Marketing Authorisation for Global Health Products (MAGHP) procedure by Swissmedic. Type 2 (abridged) is used for products previously approved by a stringent regulatory authority (SRA) and type 3 (full) is used for all major applications. All the authorities have in place a priority/fast-track procedure for applications for diseases with unmet medical need when a rapid assessment is required to obtain additional pharmacological, marketing/commercialization, pharmacovigilance, and clinical trials information.

**Table 1:** Types of review models employed by the authorities

Review model	Egypt	Ghana	Nigeria	South Africa	Tanzania	Zimbabwe
Type 1—Verification	√	√	√	√	×	√
Type 2—Abridged	√	√	×	√	√	√
Type 3A—Full	√ *	√	√	√	×	√
Type 3B—Full	√ *	×	×	√	√	×

NB: If the agency can carry out a full assessment of quality, pre-clinical (safety) and clinical (efficacy) data, then information on prior registration elsewhere may still be a prerequisite to final authorization (Model 3A) or the review may be self-standing (3B) for all major applications

*In EDA, reliance review is practiced for human pharmaceutical products through verification and abridged pathways, while reliance is practiced for biological products through two levels: reliance level 1 for products approved by the European Medicines Agency (EMA) and/or the U.S. Food and Drug Administration (FDA) with the submission of a complete unredacted assessment report from the reference agency, list of questions and answers exchanged between the applicant and the reference agency, including all annexes, a full Common Technical Document (CTD), CPP and sameness letter); and reliance level 2, which also applies to products approved by the EMA and/or FDA, however, the submission requirements include only the CTD, sameness letter, and CPP, but does not require an unredacted assessment report and list of questions and answers

A CPP (Certificate of Pharmaceutical Product) is required before local authorization by the other authorities. For the EDA, the CPP must be valid and demonstrate that product is registered and marketed in one of the 24 reference countries determined and approved by the technical committee for Drug control or WHO-Prequalified products [14]. Additionally, a complete common technical document (CTD) module is required for all models. In case of non- reference products, products undergo scientific assessment first and must obtain scientific committee approval prior to submission for registration. A letter of authorization or the detailed assessment report from the WHO-Prequalification program are, however, accepted as evidence of authorization. For SAHPRA, evidence of authorization by other countries is also accepted in place of the CPP. Additionally for type 2 reviews, the authorities refer to the public assessment reports.

### Part 3: Key Milestones in the Review Process

**Fig. 1:**
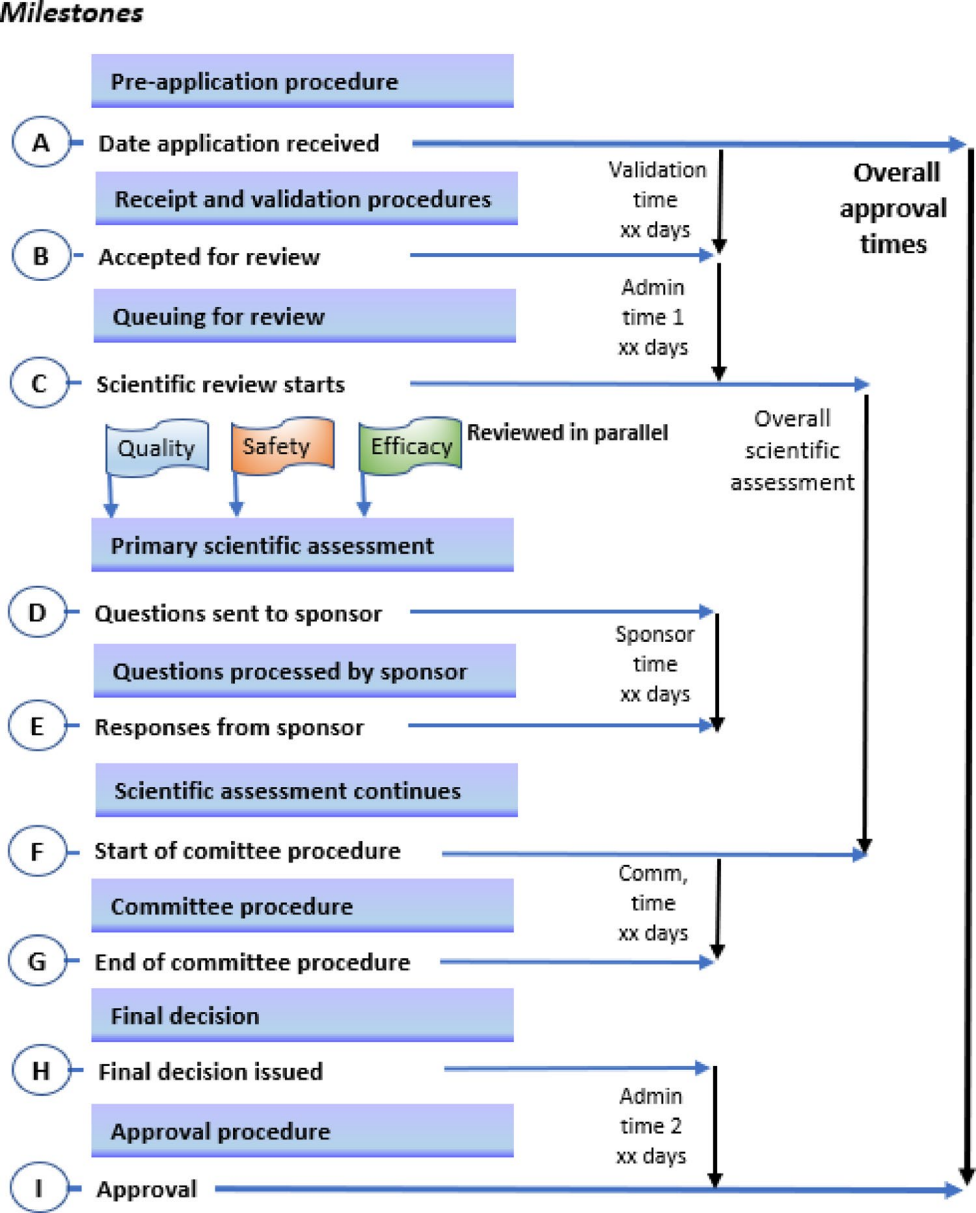
Status map of the review process and authorization of a product for a typical national medicines regulatory authority with WHO maturity level 3; with product *approved in one cycle* (that is, does not include a second or further cycles for products approved subject to the submission of additional data) and in a format that correlates with the key milestones of the review process

The authorities set targets for the time spent for review and approval (Table 2). Questions to the sponsors/applicants are batched at fixed points in the review procedure. A map of the review process and authorization of a product that is approved on the first cycle for a typical NMRA with maturity level 3 status is provided in Fig. 1 in a format that correlates with the key milestones of the review process. *Approved in one cycle* denotes that a second or further cycles were not required for products approved subject to the submission of additional data. Recording procedures allows the applicant’s response time to be measured and differentiated from the overall processing time. Generally, there is no formal procedure before the start of the application procedure. In Ghana and Nigeria some formal contact may take place during pre-submission.

**Table 2:** Comparison of authority target times in the regulatory review process

Key milestones	Egypt (working days)		Ghana (calendar days)	Nigeria (working days)	South Africa (working days)	Tanzania (working days)	Zimbabwe (calendar days)
	Human pharmaceuticals*	Biologicals*					
Receipt and validation	31	10	28	5	20	14	90
		3					
		20					
Scientific assessment	90	120	112	56	NCEs: 360; generics: 250	14	60
	198						
Applicant response time	90 renewed only once	60 renewed only once	12 months^a^	90	Clinical/quality: 30; Inspect/ naming/ sched.: 10**	180	60
Expert Committee (s)	20	20	1	30	1–2	1	N/A
Authorization procedure	10	NS	30	30	30	30	60
Overall approval time	349	173	266	120	NCEs: 472; generic: 362	240	480

N/A = Not available. NS: not specified

^a^Not later than 12, 6, and 3 months from the date of 1st, 2nd and 3rd deferrals respectively

*EDA: Normal track target timeframe for locally manufactured human pharmaceuticals and imported biological products, which dominate the Egyptian market

**Maximum 3 query rounds

### Receipt and Validation Procedures

In the first milestone for all authorities, the application is formally received and the date of receipt is recorded. The application is then checked for acceptability and completeness and if found to be satisfactory, it is accepted and then progressed to the next stage for review. The time line for this stage ranges from 5 to 90 days across the authorities (Table 2).

In the EDA, marketing authorization for human pharmaceutical and biological products falls under separate central administrations within the EDA. The Central Administration of Biological and Innovative Products and Clinical Studies for Biological Products (BioInn) handles biological products, while the Central Administration of Pharmaceutical Products (CAPP) manages human pharmaceutical products. Each administration uses its own guidelines, timeframes, and operating procedures, with some commonalities and specific differences. For both human pharmaceuticals and biological products, a registration request inquiry process is a mandatory and integral step in the marketing authorization procedure. This step serves as a prerequisite and preliminary step for the submission of the complete application and file and functions as an action letter to facilitate subsequent stages of the submission process. The inquiry process assesses the product's eligibility for registration in the Egyptian market and helps regulate the number of products available under each active ingredient. For human pharmaceuticals, the process requires 31 working days due to the substantial volume of submissions, whereas, for biological products, it is completed within 10 working days. For human pharmaceuticals, a rolling submission is implemented for the local products, allowing the incremental submission of the registration dossier to accommodate the demands of the large local market. After registration request inquiry approval, the first stage involves the submission and evaluation of naming, pharmacovigilance (PV), and pricing documents, with a target completion time of 90 working days. Upon completing this stage, the company is permitted to import raw and packaging materials for pilot batch production, enabling a six-month accelerated stability study and bioequivalence studies if required. The complete registration file must be submitted within 33 months from the approval of pricing or PV. A total of 198 days is allocated for the evaluation of the complete registration file. For biological products, the registration request inquiry takes 10 working days and this step is also responsible for approving the proposed product name and granting the applicant permission to submit the pricing file to the pricing unit within 30 days of the request inquiry approval issuance. The biological products evaluation process encompasses 3 days: assigning a meeting for file submission, 20 days for screening and validation and 120 working days for complete file evaluation.

In Nigeria, new applications are held in a queue for approximately two weeks. The authority addresses its backlog by increasing the number of assessors, workspace and other resources, developing new and transparent assessment flow charts to depict good peer-review practice as well as working on product review performance metrics versus volume of applications received to improve the efficiency of the review process. In South Africa, new applications are held in queue for approximately one year.

In Tanzania, new applications are held in a queue for approximately two to eight weeks prior to scientific assessment. To address its backlog, the authority organizes joint assessment sessions every two months in which both internal and external reviewers participate. Additionally, special sessions are organized regularly to ensure that applications are assessed on time. In Ghana, new applications are held in a queue for approximately two to six months. To address its backlog, the authority organizes assessment sessions on bi-monthly basis. In Zimbabwe, applications that have a positive outcome after screening join a queue for scientific assessments, which commences within 180 calendar days following the receipt of the application. Priority products are always taken out of the queue in all the above authorities.

### Scientific Assessment

A dossier in the CTD format, with all the five modules duly completed, is required for all types of scientific assessments in all the authorities. For a new application, the different sections of technical data (Quality, Safety, Efficacy) are reviewed in parallel. In Ghana and Nigeria, external experts are not involved with assessments, but in Tanzania, both internal and external experts carry out the scientific assessment. The timelines for scientific assessment ranges from 56 to 150 days (Table 2). Price negotiations are separated from the technical review and do not hold up the approval of products in any of the authorities.

Questions are collected into a single batch and sent to the sponsor after the initial assessment but before reporting to the Expert Committee(s). The scientific review ceases while questions are being processed by the sponsor; that is, a clock stop is applied. The timeline given to sponsors to provide responses to questions range from 30 to 180 days for all the authorities except for Ghana where applicants have 12, 6 or 3 months to respond to first, second or third deferrals respectively (Table 2). In all the authorities, applicants can hold meetings with the authority staff to discuss questions and clarify issues that arise during the assessment. Expert committees are integrated into the internal/external scientific review procedures in the authorities. In some of the authorities studied, it is mandatory to follow the committee’s recommendation whilst in other authorities, the committee acts only in an advisory capacity. The timeline for review by the expert committee ranges from 1 to 30 days (Table 2).

Authorization is not dependent on sampling analysis, although this does not apply to every application. Focus is rather on checking the product’s quality in the marketplace so that requirements for analytical work do not hold up the marketing authorization. The analytical work is started in parallel with the scientific review. In the EDA, for human pharmaceuticals, sample analysis of the first received shipment is conducted after the issuance of the final marketing authorization license. This is unlike the case for biological products for which the sample analysis before the issuance of final marketing authorization is mandated for all review types except, reliance level 1, where marketing authorization can be issued and the analysis can be deferred to the first shipment stage, prior to the product being placed on the market. For these products, conditional marketing authorization will be granted, allowing for analysis before the product’s market introduction.

Authorization is also not dependent on a pricing agreement. The EDA requires information relating to pricing as part of its review process. A separate committee carries this out and pricing submission is requested before submission of the file for validation and evaluation and pricing certificate is a request before final marketing authorization issuance.

All negotiations regarding a product’s safety, quality, and efficacy and the product information and labelling are carried out during assessment. The manufacturing facility’s compliance with current good manufacturing process (cGMP) is also considered in the marketing authorization application decision. The sponsor is not informed of a positive scientific opinion before the authorization is issued. The time for this final stage ranges from 30 to 90 days.

Table 3 shows the number of generics and WHO-prequalified medicines approved in 2023 and the mean review times from receipt of application to approval according to type of review model employed and Table 4 shows the number of new active substances and major line extensions approved from receipt of applications to approval, also according to type of review model used.

**Table 3:** Number of generics and WHO-PQ medicines approved in 2023 and mean review times from receipt of application to approval according to review model

National medicines regulatory authority	Generics approved, (n)	Mean review times	WHO-PQ approved, (n)	Mean review times
Egypt	N/A	N/A	N/A	N/A
Full				
Abridged				
Verification				
Ghana				
Full	534	56 days	0	N/A
Abridged	43	116 days	0	N/A
Verification	0	0	3	128 days
Nigeria	N/A	N/A	N/A	N/A
Full				
Abridged				
Verification				
South Africa	315 master applications		7 masters applications	
Full		240 working days		228 working days
Abridged		232 working days		167 working days
Verification		146 working days		n/a
Tanzania				
Full	359	85 days	12	79 days
Abridged	0	0	0	0
Verification	0	0	0	0
Zimbabwe				
Full	112	31 months	0	0
Abridged	40	24 months	0	0
Verification	0	0	5	10 months

WHO-PQ = WHO-prequalified

**Table 4:** Number of NASs and MLEs approved in 2023 and mean review times from receipt of application to approval according to review model

National medicines regulatory authority	NASs approved, (n)	Mean review times, days	MLEs approved, (n)	Mean review times, days
Egypt	N/A	N/A	N/A	N/A
Full				
Abridged				
Verification				
Ghana				
Full	0	N/A	0	N/A
Abridged	16	116	0	N/A
Verification	0	N/A	0	N/A
Nigeria				
Full	0	0	0	0
Abridged	1	30	18	118
Verification	0	0	0	0
South Africa	48 master applications		2 master applications	
Full		243 working days		104 working days
Abridged		102 working days		N/A
Verification		32 working days		N/A
Tanzania				
Full	0	N/A	0	N/A
Abridged	0	N/A	0	N/A
Verification	0	N/A	0	N/A
Zimbabwe				
Full	24	33 months	0	0
Abridged	3	20 months	0	0
Verification	0	0	0	0

NASs = new active substances; MLEs = major line extensions; N/A- not applicable

From the number of generics approved and mean review times reported in Table 3, all the countries except Zimbabwe met their timelines in 2023 (Table 3); however, the respective data from Egypt and Nigeria were not available.

From the number of new active substances approved and mean review times reported in Table 4, all the countries except Zimbabwe met their timelines in 2023 (Table 4); however, the respective data from Egypt and Tanzania were not available.

### Part 4: Good Review Practices

Good review practices (GRevPs) relate to measures that have been implemented in order to achieve quality, transparency, consistency, and continuous improvement initiatives in the regulatory process. The authorities in this study put a high priority on building quality into their processes and have measures in place to monitor and improve the quality, overall consistency, transparency, and predictability of the regulatory process and achieve stakeholder satisfaction. A comparison of quality measures implemented by the authorities is provided in Table 5. It was noted that the authorities have implemented almost all the measures at this time. Only SAHPRA did not specify if standard operating procedures are used for any other procedures in the regulatory review process.

**Table 5:** Comparison of quality measures implemented by the authorities

Quality measures	Egypt	Ghana	Nigeria	South Africa	Tanzania	Zimbabwe
Internal quality policy	√	√	√	√	√	√
Good review practice system	√	√ (informally implemented)	√	√	√ (informally implemented)	√ (informally implemented)
Standard operating procedures for guidance of assessors	√	√	√	√	√	√
Standard operating procedures for the product registration committee consulted during the review process	√	√	√	√	√	Not specified
Assessment templates	√	√	√	√	√	√
Assessment report	√	√	√	√	√	√
SOP for completing the assessment report	√	√	√	√	√	√
SOP for any other procedures in the regulatory review process (e.g. validation)	√	√	√	Not specified	√	√
Dedicated quality department	√	√	√	√	×	Not specified
Scientific committee	√	√	√	√	√	√
Shared and joint reviews	√*	√	√	√	√	Not specified

* In July 2023, the EDA and the South African Health Products Regulatory Authority (SAHPRA) signed a Memorandum of Understanding (MoU) to establish the EDA-SAHPRA Work Sharing Initiative (WSI) for Registration. In October 2024, both authorities extended an invitation to industry partners to participate in the pilot phase of this initiative, scheduled to commence in 2025

A comparison of transparency and communication parameters implemented by the authorities is provided in Table 6. It was noted that the authorities have implemented almost all the measures at this time although Nigeria, Tanzania, and Zimbabwe that had some remaining parameters to be implemented.

**Table 6:** Comparison of transparency and communication parameters implemented by the authorities

Parameter	Egypt	Ghana	Nigeria	South Africa	Tanzania	Zimbabwe
Feedback to industry on submitted dossiers	√	√	×	√	√	√
Details of technical staff to contact	√ (Informally)	√ (informally)	×	√	×	×
Pre-submission scientific advice to industry	√	√ (informally)	√	√ (informally)	√	√
Official guidelines to assist industry	√	√	√	√	√	√
Industry can track progress of applications	√* Manually	√	√	√ (informally)	√	√
Summary of grounds on which approval was granted	√	√	×	√	×	×
Approval times	√	√	√	√	√	√
Advisory committee meeting	√	√	√	√	√	√
Approval of products	√	√	√	√	√	√

Informally implemented = by custom and practice i.e., it has never been clearly defined or codified but over time has become the process. ×  = Not implemented

*There is no electronic tracking system for applicants to monitor their application progress. Companies can communicate with EDA staff, track their applications, and obtain updates through email, online inquiry links, internal departmental phone lines, or pre-requested in-person meetings

A comparison of continuous improvement initiatives implemented by the authorities is provided in Table 7. Authorities, with the exception of Egypt and Zimbabwe, have not implemented external peer review initiatives and only Egypt, Nigeria and Zimbabwe have implemented an internal peer review initiative.

**Table 7:** Comparison of continuous improvement initiatives implemented by the authorities

Initiative	Egypt	Ghana	Nigeria	South Africa	Tanzania	Zimbabwe
External peer review	√	×	×	×	×	√
Internal peer review	√	×	√	×	×	√
Internal tracking systems	√	×	√	√ (informally)	√	√
Review of assessors’ feedback	√	√	√	√	√	√
Review of stakeholders’ feedback	√	√	√	√ (indirectly through Industry Task Group)	√	√

×  = Not implemented. N/A = Not available

A comparison of training and continuing education as an element of quality showed that all the authorities have implemented the following; training program for assessors, internal workshops/conferences, external courses, in-house courses, on-the-job training, external speakers invited to the authority, induction training, sponsorship of post-graduate degrees and placement and secondments in other regulatory authorities.

Some of the authorities seek direct assistance of more experienced authorities in the development of standard operating procedures (SOPs) and guidelines—by using reference documents from WHO and European Medicines Agency (EMA), jointly develop and review some guidelines with the Federal Institute for Drugs and Medical Devices (BfArM), collaborate with West African Health Organization (WAHO), EAC and SADC and other authorities such as WHO, Medicines and Healthcare products Regulatory Agency (MHRA), European Directorate for the Quality of Medicines and Healthcare (EDQM) and BfArM in the training of assessors.

In addition, some of the authorities have the following in place; tools to build quality into the assessment process, internal mechanisms for quality management (internal audits and process audits), and external quality audits by an accredited certification body to improve the system. SAHPRA’s strategy is to build capacity through recruitment and training, secondments to other regulatory authorities, and joint reviews with other regulatory authorities in order carry out more of its assessments within the authority.

### Part 5: Quality Decision-Making Processes

Quality decision-making processes relate to the decision-making frameworks in place that form the basis of the decision to approve or reject a marketing authorization application and measures available to minimize the impact of subjective influences/biases on those processes. A summary of implementation of the ten Quality Decision-Making Practices (QDMPs) by the authorities is provided in Table 8. It is noted that these practices have been largely implemented into the framework of each authority. However, a formal assessment to periodically measure the quality of decision-making processes within the authority is only fully in place in Tanzania. The decision-making process of the other authorities for approving/rejecting a marketing authorization application could therefore be improved.

**Table 8:** A summary of implementation of the ten Quality Decision-Making Practices (QDMPs) by the authorities

Practice	Egypt		Ghana		Nigeria		South Africa		Tanzania		Zimbabwe	
	Implemented into framework	Adhered to in practice	Implemented into framework	Adhered to in practice	Implemented into framework	Adhered to in practice	Implemented into framework	Adhered to in practice	Implemented into framework	Adhered to in practice	Implemented into framework	Adhered to in practice
Have a systematic, structured approach	√	√	√	√	√	√	√	√	√	√	√	√
Assign clear roles and responsibilities (decision makers, advisors, information providers)	√	√	√	√	√	√	√	√	√	√	√	√
Assign values and relative importance to decision criteria	√	√	√	√	√	√	√	√	√	√	√	√
Evaluate both internal and external influences/biases	√	√	√	√	√ (partially)	√ (partially)	√	√	√	√	√	√
Examine alternative solutions	√	√	√	√	√ (partially)	√ (partially)	√	√	√	√	√	√
Consider uncertainty	√	√	√	√	√ (partially)	√ (partially)	√	√	√	√	√	√
Re-evaluate as new information becomes available	√	√	√	√	√	√	√	√	√	√	√	√
Perform impact analysis of the decision	√	√	√ (In progress)	√ (In progress)	√ (partially)	√ (partially)	Not specified	Not specified	√	√	√	√
Ensure transparency and provide a record trail	√	√	√	√	√	√	√	√	√	√	√	√
Effectively communicate the basis of the decision	√	√	√	√	√	√	√	√	√	√	√	√

### Part 6: Concluding Observations

The effectiveness and efficiency of an authority’s review procedure and decision-making processes for applications are mainly influenced by barriers and drivers. The following were identified by the authorities as key barriers: insufficient data on the product, unsatisfactory quality (chemistry, manufacturing and control) reports on the products, unsatisfactory good manufacturing practice compliance report, poor quality dossiers/regulatory submissions, inadequate number of competent assessors, lack of reliance policy and framework, slow turnaround times for recognized reference authorities to provide reports, inadequate support from industry, poor compilation of the technical information for product registration leading to consumption of considerable time for assessment., workload outweighing the available human resources, insufficient funding to support as many assessment sessions as possible and inadequacy of expertise in some areas such as biologicals.

The following key positive drivers were identified by the authorities: continuous professional training, continuous internal audit, development of published timelines, integrated quality management systems, competency of the assessors, implementation of good review practices., existence of a framework for registration of new active substances (NASs), availability of guidelines for assessors, international guidelines and templates, collaborative agreements with ZaZiBoNa, WHO and other regulatory authorities, proper compilation and correctness of technical information for product registration, timely response of queries from sponsors and independence of the authority in the review process and decision making.

## Discussion

The comparative study of the regulatory systems and practices in the NMRAs that have achieved WHO maturity level 3 status has shown that some similarities exist, all of which translate into strengths for these NMRAs. The study also highlighted various differences or gaps and, with the exception of FDA Ghana, the ability of the NMRAs to carry out their regulatory mandate autonomously. This marks an ideal starting point for them to become WHO listed authorities. The correlation between the extent of autonomy of an NMRA and its regulatory performance has been previously reported [17].

This study has revealed that the human resource capacity in each of the African NMRAs is inadequate to carry out its regulatory mandate. The benefits of having the requisite human resources for optimal regulatory activities has been well documented in the literature [15]. Generally, the assessors in the NMRAs in Africa are pharmacists; however, unlike generics, the assessment of NASs covers Module 4 of the CTD dossier and requires the involvement of toxicologists or assessors who have the requisite skills to assess preclinical data/animal studies. The number of such experts in Africa, though strongly suspected to be inadequate, is not in the public domain. This gap in human resources prolongs the timeline for assessing and registering NASs in lower- and middle-income countries (LMICs) and ultimately impedes patients’ access to some NASs, which are assessed via the full assessment pathway by the NMRAs in Africa [16, 17]. The NMRAs in Africa can learn directly from other regulatory authorities with regard to the innovative strategies that were deployed to issue timely marketing authorization for COVID vaccines during the pandemic. They may also have comparative strategies in place that would assist these NMRAs to process applications for NASs that require Africa as their gateway to the rest of the world [16, 17]. The fact that Nigeria does not use the type 2 review model and Tanzania does not use the type 1 review model may not be an issue at this time as long as the processing timelines are met for the related marketing authorization applications.

It is important to note that comparing the key stages and milestones in the review processes and authorization procedures of the NMRAs in Africa showed several similarities, typical of institutions that have attained the same maturity level. In the WHO Prequalification Team: Medicines (PQTm) procedure, review of product information is conducted in the last stage of the process prior to prequalification of a product, The rationale for reviewing the product information in the final stages of the prequalification process is two-fold; the first of which is to facilitate the preparation of the public assessment report, and the second is to ensure that the Summary of Product Characteristics (SmPC), Patient information leaflet (PIL), and product labels, which are major components of the public assessment report, reflect the final product information of the manufacturer, as approved by the authority. The approach by the WHO prequalification program facilitates timely issuance of public assessment reports [18]. The ideal practice prevents duplication of efforts and could make an NMRA efficient in allocating its resources to satisfy its stakeholders. Presently, it is only Tanzania that publishes public assessment reports, and therefore, it will be helpful for the other NMRAs to reconsider the stage at which the review of labelling information is carried out. This will help the NMRAs to publish public assessment reports in a bid to become more transparent to their stakeholders and meet an important criterion of attaining maturity level 4 [19].

To be more effective, NMRAs in Africa should institutionalize some of these additional meetings (scientific advice, early clarification, late clarification, and accelerated application hearing) with applicants in order to optimize the marketing authorization procedure. The queuing of applications in the NMRA review process is an opportunity for improvement. The NMRAs should consider learning about innovative regulatory pathways for NASs from the Republic of Korea and Singapore in order to attract new product applications, most of which are needed in Africa to address the continent’s ever-increasing health needs [12].

Regarding good review practices, the absence of external peer review initiatives should be addressed, since such initiatives help to solve the problem of capacity building of the NMRAs. The NMRAs stand to benefit from the skills and expertise of external experts when they are involved with the review process.

It is commendable to note that these maturity level-3 authorities have implemented all the training and continuing education indicators. It appears that they have adopted a benchmarking culture to continually improve their regulatory systems by incorporating lessons from other institutions such as WHO, MHRA, EDQM, and BfArM) who have been proven to be comparatively more successful in providing efficient and effective services to the public and stakeholders [1]. This culture should be encouraged as the authorities stand to benefit from such collaborations to achieve “strong, efficient and sustainable regulatory systems” [20].

From the study, Nigeria’s regulatory processes for NASs were reviewed using an abridged pathway, which demonstrated that their review time of 30 days was considerably lower than Ghana (116 days) and South Africa (102 days). The reason for this difference is due to Nigeria’s strategy to embed reliance in an efficient, structured, and systematic manner.

There were, however, some gaps observed with regard to implementation of the QDMPs by the authorities. Addressing these gaps would result in the NMRAs making progress toward the achievement of WHO GBT maturity level-4 status.

There are a number of limitations of the study. There was a lack of some data from a number of participating authorities such as number of products approved and the relevant review times. In addition, although data for Egypt were provided by the Egyptian authority, it was based on publicly available information but not through completion of the OpERA questionnaire.

### Recommendations

1. There should be collaboration amongst the NMRAs that have achieved WHO GBT maturity level-3 status. An expert working group consisting of assessors from these NMRAs can apply their relatively stringent standards in the assessment of NASs and the outcome of the assessment could be applied throughout the African continent through an innovative collaborative procedure. This collaboration will enhance access to much-needed NASs by patients in Africa.
2. A mutual recognition procedure should be established to significantly reduce duplication in assessments and use resources more efficiently.
3. The recently established AMA should engage these maturity level-3 NMRAs to explore ways that the AMA could benefit from their experience and resources, thereby supporting the effectiveness and efficiency of the AMA in achieving its overall goal.
4. More capacity-building opportunities in regulatory science including training in non-clinical toxicity should be made available to NMRAs in Africa.
5. The regulatory review process of the NMRAs in Africa should be adjusted such that review of product labelling is conducted at the end of the review process and prior to the authorization of the application to facilitate the preparation of public assessment reports.
6. Authorities should have a formal assessment to periodically measure the quality of their decision-making processes in place.
7. The NMRAs should implement the nine principles in the Good Regulatory Practices guidance document- “legality, consistency, independence, impartiality, proportionality, flexibility, clarity, efficiency and transparency- as these are relevant to all authorities responsible for the regulation of medical products, irrespective of their resources, sophistication or regulatory model”
8. The NMRAs striving to attain ML3 should consider implementing the best practices that were determined from this study.

## Conclusions

This study compared the drug regulatory systems and practices in the NMRAs in Africa that have achieved WHO maturity level 3 status. Although many similarities were observed, some differences or gaps were identified. It is hoped that the NMRAs in Africa, who have achieved maturity level 3, will build on their strengths, address the identified gaps, and implement the recommendations in this study in their WHO global benchmarking-journey to reach WHO maturity level 4. The NMRAs that are yet to attain WHO GBT maturity level 3 can benefit from the outcomes of this study by implementing the best practices identified such as capacity building and adoption of reliance pathways as well as good regulatory and quality decision- making practices. It should be recognized that implementation of the best practices listed would require planned strategy as well as appropriate monetary investment.

## Data Availability

The data are available from the authors upon reasonable request.
